# Effectiveness of preconception interventions in primary care: a systematic review

**DOI:** 10.3399/BJGP.2022.0040

**Published:** 2022-11-15

**Authors:** Nishadi N Withanage, Jessica R Botfield, Sonia Srinivasan, Kirsten I Black, Danielle Mazza

**Affiliations:** GAICD, (Graduate of the Australian Institute of Company Directors), head, Department of General Practice, Monash University, Australia.; GAICD, (Graduate of the Australian Institute of Company Directors), head, Department of General Practice, Monash University, Australia.; GP registrar, Western Health, Australia.; University of Sydney, Australia.; GAICD, (Graduate of the Australian Institute of Company Directors), head, Department of General Practice, Monash University, Australia.

**Keywords:** general practice, preconception care, pregnancy outcomes, pre-pregnancy care, primary care

## Abstract

**Background:**

Primary care-based preconception care (PCC) has the potential to improve pregnancy outcomes, but the effectiveness is unclear.

**Aim:**

To evaluate the effectiveness of primary care-based PCC delivered to reproductive-aged females and/or males to improve health knowledge, reduce preconception risk factors, and improve pregnancy outcomes.

**Design and setting:**

A systematic review of primary care-based PCC.

**Method:**

Ovid MEDLINE, Cochrane CENTRAL, Embase, Web of Science, Scopus, and CINAHL were searched for randomised controlled trials (RCTs) published between July 1999 and May 2021. Two reviewers independently evaluated article eligibility and quality.

**Results:**

Twenty-eight articles reporting on 22 RCTs were included. All but one focused on females. Interventions included brief education (single session) (*n* = 8), intensive education (multiple sessions) (*n* = 9), supplementary medication (*n* = 7), and dietary modification (*n* = 4). Brief education improved health knowledge in females (*n* = 3) and males (*n* = 1), reduced alcohol/ tobacco consumption (*n* = 2), and increased folate intake (*n* = 3). Intensive education reduced spontaneous pregnancy loss (*n* = 1), alcohol-exposed pregnancies (*n* = 2), and increased physical activity (*n* = 2). Supplementary medication increased folate intake (*n* = 4) and dietary modification reduced pre-eclampsia (*n* = 1) and increased birth weight (*n* = 1). Only eight articles reported on pregnancy outcomes, with a range of interventions used; of these, four reported improvements in pregnancy outcomes. Most RCTs were of low quality (*n* = 12).

**Conclusion:**

Primary care-based PCC including brief and intensive education, supplementary medication, and dietary modification are effective in improving health knowledge and reducing preconception risk factors in females, although there is limited evidence for males. Further research is required to determine whether primary care-based PCC can improve pregnancy outcomes.

## INTRODUCTION

Preconception care (PCC) refers to interventions delivered before conception that modify preconception risk factors and reduce the burden of adverse pregnancy outcomes such as low birth weight, spontaneous abortion, and preterm birth.^1^^,^^2^ These interventions may take the form of preconception counselling or education, dietary modification, and supplementary medication during the preconception period.^1^^–^4 Previous systematic reviews have shown that PCC interventions provided in hospital and community settings improve pregnancy outcomes^4^^–^6 and health knowledge,^7^ and reduce preconception risk factors. However, less is known regarding the effectiveness of primary care-based PCC.^8^

As the first point of healthcare contact, primary care providers are ideally placed to provide PCC; however, the effectiveness of primary care-based PCC interventions is unclear.^9^^–^11 PCC is often a low priority and not routine practice in primary care in many countries^1^^,^^8^^,^^9^^,^^12^ and almost all primary care-based PCC interventions are directed towards women.^8^^–^10^,^^12^^–^18

As modifiable risk factors including smoking and alcohol consumption may also have an impact on men’s reproductive health^19^ and sperm quality,^20^ PCC directed towards reproductive-aged males may also improve pregnancy outcomes. Since the previous review investigating the effectiveness of primary care-based PCC interventions,^8^ a number of studies evaluating the effectiveness of PCC interventions in primary care have been published. In the current study therefore a systematic review was conducted to evaluate the effectiveness of primary care-based PCC interventions delivered to reproductive-aged females and/or males to improve health knowledge, reduce preconception risk factors, and improve pregnancy outcomes. This builds on the previous review published in 2016 that was limited to females, and which included randomised controlled trials (RCTs) published between July 1999 and July 2015.^8^

## METHOD

### Search strategy and selection criteria

The Preferred Reporting Items for Systematic Review and Meta-Analysis Protocols (PRISMA-P) guidelines were followed^21^ and the review was registered with PROSPERO (CRD42021235499) as described in the protocol.^22^ Search terms were developed (Supplementary Table S1) and Ovid MEDLINE, Cochrane CENTRAL, Embase, Scopus, CINAHL, and Web of Science were searched. The pilot search for this review showed heterogeneity of outcomes measured across the studies, therefore search terms relating to outcomes were not included in the search strategy. Reference lists of included articles were manually screened for additional studies. Articles were included if the study:

- reported on the effectiveness of PCC in primary care;
- included reproductive-aged males and/ or females (18–45 years);
- was an RCT;
- was written in English;
- was published in a peer-reviewed journal between July 1999 and May 2021; and
- included but was not limited to the outcomes outlined in Box 1.

**Box 1.** Population, Intervention, Control and Outcomes (PICO) criteria and search terms

Population terms	teen* or adolescen* or youth or men or man or female or male or woman or women or reproductive age or child bearing age or childbearing age
Intervention terms	preconcept* or pre concept* or interconcept* or prepregnan* or pre pregnan* or pregnanc* plan* or plan* pregnanc* and health program* or health education or health promot* or advic* or advis* or intervention* or care or assess* or risk or counsel* or screen* or folic acid supplement* or folate supplement*
Control	No preconception care/usual care
Outcomes	Primary outcomes included but not limited to pregnancy outcomes including maternal morbidity, prematurity, birth weight, fetal/neonatal mortality, morbidity, fetal abnormalities, and/or health knowledge of preconception risk factors Secondary outcomes included reduction in modifiable preconception risk factors including but not limited to alcohol consumption, smoking, folate deficiencies, maternal mental health conditions, obesity and/or drug use
Limits and restrictions	English language only Randomised control trials only, as they are the reference standard for studying causal relationships between interventions and outcomes^24^ July 1999 to May 2021 Human studies

**Table:** How this fits in

Preconception care (PCC) delivered in community and hospital settings are effective in improving pregnancy outcomes and health knowledge, and reducing preconception risk factors; however, the effectiveness of primary care-based PCC has been unclear. This systematic review demonstrates that primary care-based PCC including brief and intensive education, supplementary medication, and dietary modification are effective in improving health knowledge and reducing preconception risk factors among females, even when delivered by trained non-healthcare professionals. Non-healthcare professionals could help improve access to PCC in systems that are already struggling to provide care. As there is a limited number of studies reporting on pregnancy outcomes, further research is required to determine whether primary care-based PCC can improve pregnancy outcomes.

Articles were excluded if the study: included pregnant females or focused on fertility. The start date was selected following the end of the search of an earlier review by Korenbrot *et al*.^23^ Only RCTs were included as they are the reference standard for studying causal relationships between interventions and outcomes.^24^

All results of database searches were saved in Covidence systematic review software, Veritas Health Innovation, Melbourne, Australia for management. Duplicates were removed and two authors independently screened all titles, abstracts, and full texts of articles for eligibility; discrepancies were reviewed by a third author to reach consensus. Two authors independently evaluated the quality of the RCTs using the Cochrane risk-of-bias (ROB 2.0)^25^ tool with six domains: sequence generation, allocation concealment, blinding of participants and personnel, blinding of outcome assessors, incomplete outcome data, and selective reporting bias. Studies were classified as high quality (low risk-of-bias for all domains), moderate quality (if at least one domain is unclear, but not at high risk-of-bias for any domain), or low quality (high risk-of-bias in at least one domain or unclear risk-of-bias in multiple domains).

### Data extraction and analysis

One of the authors created a data extraction form based on previous reviews on PCC^7^^,^^8^ and extracted data (Supplementary Table S2).^8^^,^^12^^,^^18^ The pilot search for this review also showed that meta-analysis cannot be undertaken because of the heterogeneity of the outcomes investigated across the different studies. The magnitude of the difference between the control and intervention groups are presented as reported in the RCTs. For dichotomous data, relative risks, odds ratios (OR) with 95% confidence intervals (CI), are presented and for continuous data the mean difference before and following the intervention and/or *P*-values are reported.

## RESULTS

**Figure 1.**
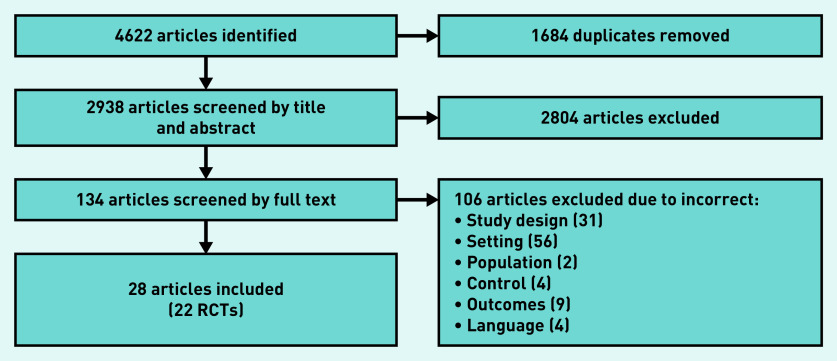
*PRISMA flow diagram for papers included in the review. RCT = randomised controlled trial.*

Out of 4622 articles, 1684 duplicates were removed and 134 full-text articles were evaluated for eligibility after title and abstract screening. After full-text screening, 28 articles were included, reporting on 22 RCTs (Figure 1). No additional articles were identified through manually screening reference lists of included articles. The included articles were from the US (*n* = 8),^26^^–^33 Iran (*n* = 8),^34^^–^41 Vietnam (*n* = 5),^42^^–^46 China (*n* = 2),^47^^,^^48^ the Netherlands (*n* = 2),^49^^,^^50^ India (*n* = 1),^51^ Australia (*n* = 1),^52^ and Sweden (*n* = 1).^53^ Studies recruited females who were: aged 18–35,^40^ aged 18–45,^26^^,^^30^ able to conceive,^28^^,^^33^^,^^37^ planning to have children,^41^^,^^47^^,^^48^^,^^51^ planning pregnancy within 9 months,^27^ a year,^31^^,^^35^^,^^36^^,^^38^^,^^39^^,^^43^^,^^44^^,^^46^ 2 years,^29^ 1–5 years,^48^^,^^49^ or after the delivery of their first baby,^32^^,^^42^^,^^45^^,^^52^ and one study recruited males.^53^ The majority recruited females planning for pregnancy (*n* = 15).^27^^,^^29^^,^^30^^,^^35^^,^^36^^,^^38^^,^^39^^,^^43^^,^^44^^,^^46^^–^50^,^^51^ The type of intervention(s), provider, and outcome(s) measured varied across studies. In some studies the providers were healthcare professionals^26^^,^^30^^,^^31^^,^^43^^,^^44^^,^^46^^–^50^,^^51^^–^53 whereas in other studies non-healthcare professionals^27^^–^29^,^^32^^–^42^,^^45^ were trained to deliver the intervention (the latter referred to hereafter as ‘trained facilitators’).

The PCC interventions were categorised into brief education (a single education session),^26^^,^^35^^–^37^,^^49^^,^^50^^,^^52^^,^^53^ intensive education (multiple education sessions),^27^^–^29^,^^33^^,^^34^^,^^38^^–^41 supplementary medication,^43^^,^^44^^,^^46^ dietary modification,^42^^,^^45^^,^^48^^,^^51^ and multiple interventions.^30^^–^32^,^^47^ Studies reported on the effectiveness of the intervention on pregnancy outcomes,^29^^,^^42^^,^^44^^–^46^,^^48^^,^^50^^,^^51^^,^^52^ health knowledge,^37^^,^^38^^,^^50^^,^^53^ and/or preconception risk factors.^25^^–^28^,^^30^^–^37^,^^39^^–^41^,^^43^^,^^47^^,^^49^ Study characteristics and quality are summarised in Supplementary Tables S2 and S3. According to the ROB 2.0^25^ tool, only two of the RCTs were of high quality (*n* = 2, 9%), eight were of moderate quality (*n* = 8, 36%), and the majority were of low quality (*n* = 12, 55%).

### Effectiveness of brief education

Eight articles reported on the effectiveness of brief education (a single session).^26^^,^^35^^–^37^,^^49^^,^^50^^,^^52^^,^^53^ In four of these, healthcare professionals, including GPs,^49^^,^^50^ nurse–midwives,^52^ and midwives, directed the sessions.^53^ The other four were directed by non-healthcare professionals, including trained facilitators^26^^,^^35^^,^^36^ or the researcher.^37^ Healthcare professional-directed brief education improved pregnancy outcomes in one of two studies investigating this,^50^^,^^52^ improved health knowledge, and reduced preconception risk factors in females^49^^,^^50^ and males.^53^

In the one study that reported improved pregnancy outcomes, GP-directed counselling about preconception risk factors decreased adverse pregnancy outcomes including miscarriage, extra-uterine pregnancy, perinatal death, preterm birth, low birth weight, and congenital abnormalities.^50^ This study also reported increased self-reported folate intake (OR 4.93, 95% CI = 2.81 to 8.66)^50^ and reduced alcohol use during the first trimester (OR 1.79, 95% CI = 1.08 to 2.97), and self-reported maternal anxiety (OR not reported).^49^ In the second study, midwife-directed counselling during a home visit did not affect the incidence of preterm birth or low birth weight during subsequent pregnancies.^52^

Among the 22 RCTs, only one study involved males.^53^ In this RCT, reproductive life plan-based (RLP) counselling by nurse–midwives increased men’s self-reported awareness of preconception lifestyle risk factors 3 months post-intervention (*P*<0.0001).^53^ The RLP tool provides individuals with information concerning reproductive health. After RLP counselling 76% of males in the intervention group reported a positive experience of the counselling, and 77% had received new information.

Non-healthcare professional-directed brief education reduced preconception risk factors in females in the four studies investigating this.^26^^,^^35^^–^37 Three studies reported on the effectiveness of trained facilitator-directed group workshops on preconception health and folate-focused education.^26^^,^^35^^,^^36^ Of these, group workshops in one study had a positive impact on healthy behaviours such as increasing physical activity (*P*<0.01)^36^ and the other two reported increased self-reported intake of folate-rich food.^26^^,^^35^ Likewise, a researcher-directed 5–10 min RLP counselling session increased self-reported knowledge of folate intake (*P*<0.001).^37^

### Effectiveness of intensive education

Nine articles reported on the effectiveness of intensive education.^27^^–^29^,^^33^^,^^34^^,^^38^^–^41 In all of these, sessions were directed by non-healthcare professionals, including trained facilitators^27^^–^29^,^^33^^,^^34^^,^^38^^–^41 and the researcher.^41^

Non-healthcare professional-directed intensive education improved health knowledge,^38^ reduced spontaneous pregnancy loss,^29^ or reduced preconception risk factors in all studies investigating this.^27^^–^29^,^^33^^,^^34^^,^^39^^–^41 In one study, group counselling sessions on preconception risk factors increased self-reported health knowledge of preconception lifestyle risk factors (mean difference 7.8, 95% CI = 8.7 to 6.9).^38^ In another, weekly counselling sessions on health responsibility for 6 months, followed by monthly sessions until delivery, reduced spontaneous pregnancy loss (OR 0.39, 95% CI = 0.16 to 0.92) and increased self-reported weight loss before pregnancy (*P*<0.001).^29^

A 14-week counselling programme around hazardous drinking reduced alcohol-exposed pregnancies at 3-, 6-, and 9-month follow-ups.^27^ Similarly, two motivational interviewing sessions that aimed to increase participants’ commitment to change hazardous alcohol use reduced risk of alcohol-exposed pregnancies across 9 months (incidence rate ratio 0.620, 95% CI = 0.511 to 0.757).^33^

Six 2-hour sessions on preconception risk factors also increased self-reported physical activity (*P* = 0.019).^28^ In another study, 6-weekly motivational interviewing sessions increased self-reported moderate (*P* = 0.01) and vigorous (*P* = 0.02) physical activity,^40^ and increased self-reported weight loss post-intervention (mean difference −1.457 kg, 95% CI = 2.061 to 0.852).^34^ Two studies investigated the short-term effect of preconception risk factor counselling on maternal stress levels.^39^^,^^41^ In one study, trained facilitator-directed counselling reduced self-reported maternal stress 4 and 8 weeks post-intervention.^41^ In the other, researcher-directed counselling improved stress management 1 month post-intervention.^41^

### Effectiveness of supplementary medication

Three articles from one RCT reported on the effectiveness of supplementary medication delivered by village health workers.^43^^,^^44^^,^^46^ Women receiving multiple-micronutrients (multi-micronutrient or iron and folate supplements) or iron were compared with females receiving folate only (control). Preconception multiple micronutrient supplementation did not affect the prevalence of low birth weight or preterm birth,^46^ but a follow-up analysis reported high prenatal and postpartum maternal ferritin levels in the groups supplemented with multiple-micronutrients or iron and folate and these females gave birth to infants with greater iron stores. However, the clinical significance is unclear, as anaemia prevalence did not differ between groups.^44^

Furthermore, maternal depressive symptoms were low during pregnancy and early postpartum, and there was no difference between the groups. Although the underlying mechanisms are unclear, among females at risk of depression, maternal depressive symptoms were lower in the first and second trimesters of pregnancy in the groups receiving multiple-micronutrients or iron and folate compared with the control group (*P*<0.05).^43^

### Effectiveness of dietary modification

Four articles reported on the effectiveness of dietary modification.^42^^,^^45^^,^^48^^,^^51^ In two of these, healthcare professionals including health workers^51^ or specialists^48^ delivered the intervention and improvements in pregnancy outcomes were reported. 48^.^^51^ In one study, health worker provision of a snack (made from leafy green vegetables, fruit, and milk), provided from >90 days before pregnancy until delivery of baby, in addition to their usual diet, increased infant birth weight (*P* = 0.046).^51^ This may have resulted from higher micronutrients, energy, and/or protein levels in the snack provided to the intervention group when compared with the control group.^51^ Additionally, specialist provision of a diet comprising at least 100 g of mushrooms daily from preconception to the 20th week of gestation reduced gestational hypertension (*P* = 0.023), preeclampsia (*P* = 0.014), gestational weight gain (*P* = 0.017), excessive gestational weight gain (*P* = 0.032), and gestational diabetes (*P* = 0.047).^48^ In the other two articles, non-healthcare professional trained facilitators^42^^,^^45^ delivered macronutrient supplementation from preconception to term. This increased maternal protein, iron, zinc, folate, vitamin A, and B12; however, it did not affect infant birth weight^42^ or infant growth up to 24 months of age.^45^

### Effectiveness of multiple interventions

Four studies reported on the effectiveness of multiple interventions including supplementary medication such as folate supplementation along with brief^30^^–^32 or intensive^47^ education. Interventions involved provision of folate education via a 15-min GP computerised session,^31^ brief counselling by a gynaecologist,^30^ monthly counselling by village doctors,^47^ or brief counselling by paediatric clinicians.^32^ All studies reported improvements in self-reported folate intake^30^^–^32^,^^47^ and one study reported reduction in self-reported binge drinking and smoking.^33^

## DISCUSSION

### Summary

To the authors’ knowledge, this is the first systematic review of primary care-based PCC that includes males and also the first to consider the role of the provider in the delivery of primary care-based PCC. Results from 28 articles reporting on 22 RCTs were included incorporating an additional 17 articles published since the last review.^8^ Most articles in the current review were of low quality and the type of interventions, populations, providers, and outcomes varied substantially between the different studies.

A number of important findings were identified in this review. First, both brief and intensive education on preconception health improved health knowledge and reduced preconception risk factors for females, suggesting either method could be utilised by primary care providers to deliver PCC education. However, the duration of brief education (that is, 5–10 mins, 1 hour, 1 day), intensive education (that is, undertaken over 6 weeks, 14 weeks, 18 months), and the timing of PCC education delivery (that is, 9 months, a year, 2 years, 1–5 years before conception or after the delivery of first baby) varied considerably between studies, so it is not clear which are the most effective.

Second, dietary modification improved pregnancy outcomes by reducing pre-eclampsia^48^ and increasing birth weight^51^ in two studies;^48^^,^^51^ however, the studies were of moderate to low quality and more high-quality evidence is required. Multiple interventions including brief or intensive folate education along with folate supplementation increased self-reported folate intake in all studies investigating this,^30^^–^32^,^^47^ reiterating that primary care providers should encourage supplementary medication, including folate supplements, and intake of folate-rich foods, to all females during the preconception period.

Third, although findings suggest that brief education improves health knowledge among males, more research is required as this is based on only one study. Fourth, although in 10 of the studies the intervention was delivered by healthcare professionals, in the majority of studies (*n* = 12) the intervention was delivered by non-healthcare professionals. In almost all (*n* = 11) of these latter studies, improved health knowledge,^38^ reduced preconception risk factors,^26^^–^28^,^^33^^–^36 or reduced spontaneous pregnancy loss were reported.^29^ These results suggest that primary care-based PCC are effective in improving health knowledge and reducing preconception risk factors; trained facilitators could help improve access to PCC in systems that are already struggling to provide care. Finally, although eight studies were found that reported on pregnancy outcomes, only four reported improvements.^29^^,^^44^^,^^48^^,^^50^ It is unclear whether this is related to the strength of the intervention being delivered or the intervention itself, therefore more evidence is required to understand the effectiveness of primary care-based PCC on improving pregnancy outcomes.

### Strengths and limitations

This review was not restricted to a particular region or country, and therefore provides a broad international perspective on the effectiveness of primary care-based PCC interventions. Five databases were systematically searched for literature; however, relevant articles may still have been missed because of the search strategies employed. By limiting the eligibility criteria to RCTs, non-RCTs investigating primary care-based PCC were excluded that may have reported improved pregnancy outcomes.

### Comparison with existing literature

This review included 17 additional articles published since the review published in 2016^8^ and 24 additional articles since the last Cochrane review published in 2009.^18^ Similar to another review of PCC in community settings,^4^ the current review found that primary care-based PCC interventions are effective in improving health knowledge, increasing here folate intake, and reducing alcohol consumption.

### Implications for research and practice

Given the effectiveness of PCC education delivered in primary care at reducing risk factors, brief or intensive PCC education should be mainstreamed for reproductive-aged females and males in primary care.

None of the RCTs in this review targeted PCC for females at high risk of poor pregnancy outcomes based on pre-existing^54^ medical and lifestyle health indicators. Also there is a lack of understanding about how high-risk females can be systematically identified in primary care. Research investigating how to best identify these females in primary care is therefore warranted.

Lastly, in all studies providing intensive PCC education, interventions were delivered by trained facilitators or researchers. Future studies could explore the role and potential impact of primary care providers, including GPs, nurses and midwives, and trained non-healthcare professionals delivering intensive PCC education.

In conclusion, primary care-based PCC including brief/intensive education, supplementary medication, and/or dietary modification improved health knowledge and reduced preconception risk factors among females, irrespective of the provider. Brief education may also improve health knowledge in males, although more research is needed.

Given the limited number of studies reporting on pregnancy outcomes, further research is required to determine whether primary care-based PCC is effective in improving pregnancy outcomes.
